# Genetic Variation at Nuclear Loci Fails to Distinguish Two Morphologically Distinct Species of *Aquilegia*

**DOI:** 10.1371/journal.pone.0008655

**Published:** 2010-01-19

**Authors:** Elizabeth A. Cooper, Justen B. Whittall, Scott A. Hodges, Magnus Nordborg

**Affiliations:** 1 Department of Molecular and Computational Biology, University of Southern California, Los Angeles, California, United States of America; 2 Biology Department, Santa Clara University, Santa Clara, California, United States of America; 3 Department of Ecology, Evolution, and Marine Biology, University of California Santa Barbara, Santa Barbara, California, United States of America; 4 Gregor Mendel Institute of Molecular Plant Biology, Vienna, Austria; McGill University, Canada

## Abstract

*Aquilegia formosa* and *pubescens* are two closely related species belonging to the columbine genus. Despite their morphological and ecological differences, previous studies have revealed a large degree of intercompatibility, as well as little sequence divergence between these two taxa [1], [2]. We compared the inter- and intraspecific patterns of variation for 9 nuclear loci, and found that the two species were practically indistinguishable at the level of DNA sequence polymorphism, indicating either very recent speciation or continued gene flow. As a comparison, we also analyzed variation at two loci across 30 other *Aquilegia* taxa; this revealed slightly more differentiation among taxa, which seemed best explained by geographic distance. By contrast, we found no evidence for isolation by distance on a more local geographic scale. We conclude that the extremely low levels of genetic differentiation between *A. formosa* and *A.pubescens* at neutral loci will facilitate future genome-wide scans for speciation genes.

## Introduction

The genetic mechanisms underlying the process of speciation are of critical interest to evolutionary biologists. In order to unravel this process, it is necessary to both identify the genes responsible for existing reproductive barriers and to consider what demographic and selective forces have shaped these traits. In particular, many recent studies have focused on the role of gene flow during the speciation process [3]–[10], even though the more traditional (allopatric) view of speciation posits that genetic exchange must be rare in order for species to remain distinct [11]. These studies have shown that adaptive differences between species can be maintained even in the face of significant amounts of introgression, especially if only a few genes or genomic regions control the traits that lead to reproductive isolation [4]. Genome-wide analyses of many species have shown that levels of introgression can vary across the genome, with divergent selection playing an active role in preventing gene flow at the loci underlying adaptive traits, but not acting at other areas in the genome [3], [4], [12]. Incipient species will also show varying levels of differentiation across the genome, with the most differentiated regions also being the most likely to contain genes that restrict random mating [3], [7]. These species can appear almost identical at many loci, even in the complete absence of genetic exchange.

Whether or not gene flow is a factor, closely related taxa offer an excellent opportunity to study the genetic changes and processes that lead to reproductive isolation, since genome-wide scans should be able to pinpoint loci with higher levels of differentiation, and these loci are most likely to be under the influence of natural selection [3], [13]–[15]. While the identification of potential speciation genes will not definitively prove a particular speciation model, comparing the pattern of variation in these loci with the pattern of shared variation in neutral loci will provide much more insight into the question of whether or not two species have diverged in the face of gene flow [3], [7]. Thus, it is especially important to identify pairs or groups of species that maintain high levels of shared polymorphism over much of their genomes.

The columbine genus *Aquilegia* [Ranunculaceae] is an excellent example of a recent, rapid adaptive radiation [1], and thus should provide an opportunity to identify the genetic changes important for speciation. The genus is comprised of approximately 70 outcrossing species that occupy a wide variety of habitats in North America, Europe, and Asia [16] and that differ substantially in floral morphology [16], [17]. Despite these differences, species are usually cross-compatible [18], [19].

**Figure 1:**
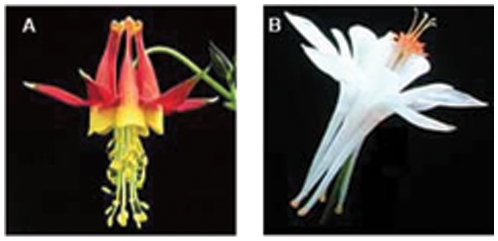
Striking differences in floral morphology between (A) the hummingbird pollinated *Aquilegia formosa* and (B) the hawkmoth pollinated *Aquilegia pubescens*.

Two species, *Aquilegia formosa* and *A. pubescens*, have long been studied for the purpose of understanding the factors controlling reproductive isolation between them [20]–[23]. *A. formosa* is found throughout mountainous regions of western North America while *A. pubescens* is restricted to the southern Sierra Nevada range [22]. The species exhibit distinct differences in floral characters that have been shown to influence pollinator preference, thereby restricting gene flow between them [22], [23](Figure 1). Additionally, they prefer different habitats: *A. formosa* populations typically occur in moist areas with well-developed soils at lower elevations (below 3,000m), whereas *A. pubescens* populations are found in drier, poorly developed soils at higher elevations (3,000–4,000m) [20], [21], [24]. However, the two species are highly interfertile, and form natural hybrid zones at mid elevations where the two habitats co-occur [22]. Molecular markers exhibit more introgression than morphological characters near these zones, suggesting that gene flow could be extensive between these species for neutral markers [22].

Previous studies have uncovered limited DNA sequence variation between *A. formosa* and *A. pubescens* in both chloroplast and nuclear sequences [1], [2]. However, these previous studies showed either low sequence variation across a wide range of *Aquilegia* species [1] or few individuals were sampled [2] and therefore do not address the degree of genetic differentiation between these species. Other studies suggest that intraspecific sequence variation may be quite similar in *A. formosa* and *A. pubescens* and thus that they may be especially useful for identifying speciation genes. For instance, microsatellite loci have similar numbers of alleles and size ranges [25], and another study including over 850 AFLP markers polymorphic in a small sample of both species found only one marker that showed complete differentiation [17]. Because these previous studies did not assess variation at the DNA sequence level or use relatively large population samples, we sought to gain insight into the inter- and intraspecific patterns of genetic variation in these species by sequencing nine nuclear loci from a total of 80 individuals from several populations. As a comparison, we also assessed variation among all of the North American species in the genus (plus some Eurasian taxa) by sequencing two of the nine nuclear loci. By examining loci that are not believed to be involved in the maintenance of reproductive isolation, we sought to assess the potential of using genome-wide scans for speciation genes in these species by determining levels of neutral variation and population structure.

## Results

### Polymorphism Levels and Linkage Disequilibrium

**Figure 2:**
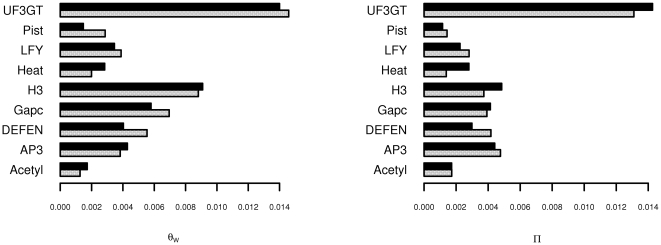
Variation among levels of polymorphism for each species. The sequences of each of the 9 fragments were grouped according to species, and 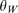 and 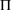 were estimated separately for each group. Grey bars represent estimates for *A. formosa*, and black bars represent *A. pubescens*.

The counts of segregating sites found in each fragment are given in Table 1. Estimates of both 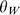 and 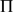 generally fell in the range of 0.004 to 0.006 per base pair (Figure 2). Overall, these estimates are slightly lower than estimates of 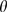 in other outcrossing plant species such as maize (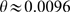) [26] and sunflowers (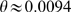) [27], similar to estimates in the model species *Arabidopsis thaliana* [28], and higher than estimates found in soybeans (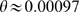) [29]. However, values of 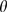 and 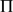 varied across the 9 fragments, and one of them, *UF3GT*, was substantially more polymorphic than the others, with a value of 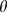 between 0.01 and 0.02 (Figure 2). The estimates of 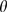 for all fragments are strikingly correlated across species; as we shall demonstrate in the next section, this is because almost all variation is shared.

**Table 1:** Polymorphism Counts for Each Fragment For each count in bold, the number of sites represents the number of SNPs plus the number of indels treated as single SNPs. Numbers in parentheses represent the number of sites with a Minor Allele Frequency (MAF) 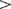
5% and 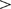
10%, respectively.

Fragment	Total 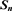	Indels	*A. formosa*	*A. pubescens*	Shared	Fixed Differences
			Exclusive	Exclusive		
*Acetyl*	**5** (2, 2)	0	**1** (0, 0)	**2** (0, 0)	**2** (2, 2)	0
*DEFEN*	**20** (8, 6)	3	**9** (1,0)	**4** (0, 0)	**7** (7, 6)	0
*Gapc*	**44** (14, 8)	14	**18** (1,0)	**11** (0,0)	**15** (13, 8)	0
*H3*	**18** (7, 2)	2	**5** (0, 0)	**5** (1, 0)	**8** (6, 2)	0
*Heat*	**20** (7, 4)	1	**7** (0, 0)	**9** (4, 1)	**4** (3, 3)	0
*AP3*	**29** (16, 12)	10	**10** (4, 3)	**11** (4, 2)	**8** (8, 7)	0
*LFY*	**12** (4, 2)	4	**6** (2, 0)	**3** (0, 0)	**3** (2, 2)	0
*Pist*	**15** (8, 4)	2	**10** (4, 1)	**2** (1, 0)	**3** (3, 3)	0
*UF3GT*	**34** (19, 16)	4	**9** (2, 0)	**6** (0, 0)	**19** (17, 16)	0

Linkage disequilibrium (LD) was not extensive in any of the 9 regions that were sequenced, with average 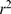 values ranging between 0.1 and 0.2 for most of the fragments. When values of 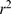 are plotted against physical distance between SNPs, the relationship is weak (Figure S7). The fragments with the highest levels of polymorphism show evidence for a rapid decay of LD (within about 1 kb or less). The combined fragment data show low LD values overall, and our estimate of 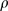 was 0.009, which is higher than estimates of 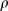 in humans [30], suggesting a relatively high rate of recombination in these species (Table 2).

**Table 2:** Estimates of recombination rate for each fragment.

Fragment Name	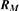	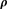
Acetyl	1	0.081
AP3	11	0.003
Defen	8	0.133
Gapc	12	0.004
H3	3	0.00
Heat	6	0.126
LFY	4	0.006
Pist	4	0.001
UF3GT	15	0.023
Combined		0.009

### Genetic Differentiation

**Figure 3:**
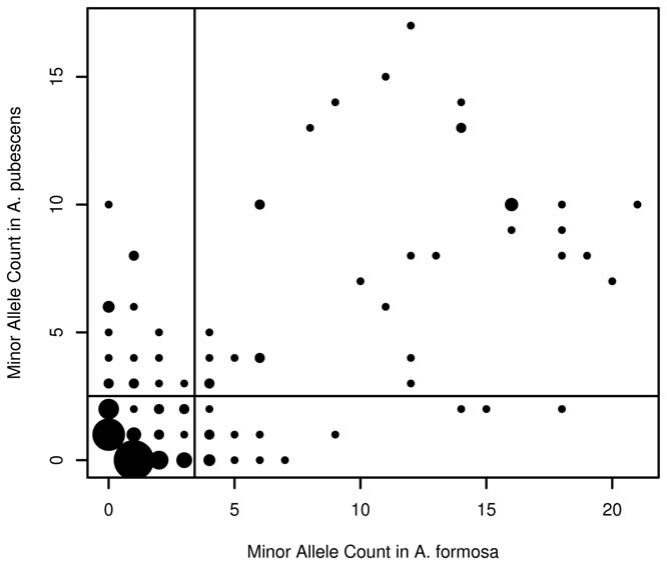
Comparison of minor allele counts in *A. formosa* and *A.pubescens*. The horizontal line represents the mean allele count in *A. pubescens*, while the vertical line represents the mean allele count in *A. formosa*. Point size reflects the number of comparisons at that point. Species-specific polymorphisms correspond to the points along either the very bottom or the far left of the plot. All other sites correspond to a shared polymorphism. There are no fixed differences. The average minor allele frequency for any species-specific polymorphism was 0.105, while the average frequency for any shared allele was 0.424.

When we compared the minor allele counts for each species, we found that few high frequency SNPs corresponded to species-specific polymorphisms (Figure 3 and Table 1). In fact, at sites with a minor allele frequency 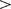
5%, there were more than twice as many shared polymorphisms (61) as species-specific polymorphisms (24). We found no fixed differences in any of the 9 sequences.

STRUCTURE was unable to cluster individuals according to species under the naïve assumption of K = 2 (Figure S1). The most likely number of clusters appeared to be around 11 (Figure S2), based on when the estimated probability and the average clusteredness stopped (consistently) increasing (Figures S3 and S4). Although the pattern of clustering does not correspond perfectly to the sample populations, it does not seem to be entirely random, especially among the more well-defined clusters (where individuals tend to have membership coefficients 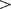
0.5). We found that pairs of individuals from the same population tended to cluster together 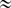
15% of the time, whereas pairs of individuals from different populations only clustered together 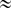
9.5% of the time (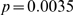 in 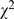 test). Similarly, 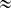
11% of same-species pairs were found in the same cluster, whereas only 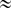
8% of different-species pairs were clustered together (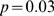).

Average 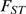 between the two species was approximately 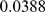 (with 95% C.I. between 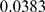 and 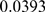), which is low, but statistically different from zero. When populations were randomly assigned to 2 groups (regardless of species), we achieved very similar results: a mean 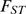 of 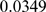 with a 95% C.I. between 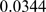 and 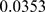. Although these estimates are technically statistically different, they do not suggest that much of the observed differentiation is due to species differences.

**Figure 4:**
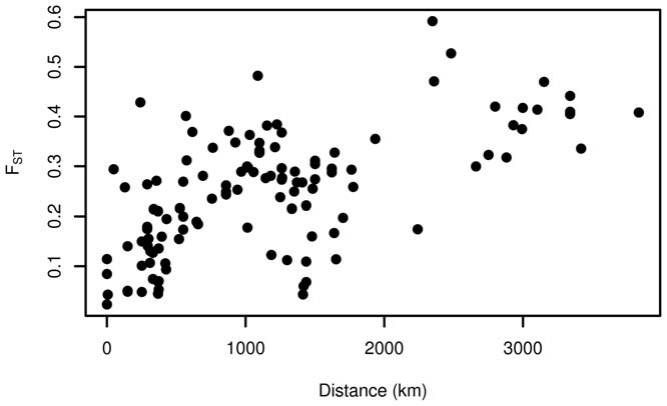
Relationship between geographic distance and genetic distance. Each dot represents a comparison between 2 populations of at least 5 individuals. For populations where there were more than 5 individuals, estimates of 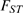 were bootstrapped to ensure that the larger sample size did not cause any bias in the estimate.

**Figure 5:**
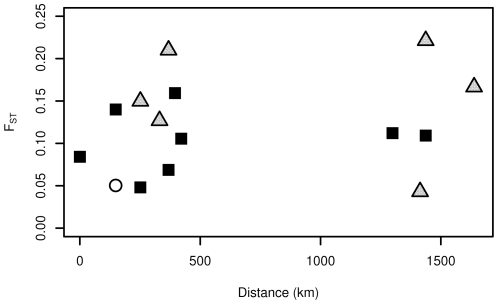
Relationship between geographic distance and genetic distance for *A. formosa* and *A.pubescens* only. Black squares represent comparisons between *A. formosa* and *A. pubescens* populations; gray triangles are comparisons among populations of *A. formosa*; white circles are comparisons among populations of *A. pubescens*.

In order to determine whether such a high degree of shared polymorphism was common in the *Aquilegia* genus or unique to *A. formosa* and *A. pubescens*, we also calculated 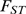 in a broader sample of 32 taxa using two gene regions (*Gapc* and *UF3GT*). We estimated 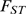 between pairs of populations and obtained a mean estimate of 0.247. Because the sample of 32 taxa encompassed a broader geographical range, we tested the relationship between geographic distance and genetic differentiation across pairs of populations (Figure 4). Results of the Mantel test indicated that geographic distance had a significant relationship with genetic differentiation within the *Aquilegia* genus (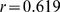, 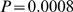)-more so than any of the other factors we examined (Figure S5). However, on a more local scale, we do not find evidence for isolation by distance either within on between *A.formosa* or *A. pubescens* populations (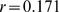, 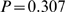) (Figure 5).

### Isolation-Migration

When MIMAR was run with the migration rate fixed at 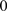, the time since the split between *A. formosa* and *A. pubescens* is estimated as approximately 0.062 in coalescent time units (Figure S6). If we assume the mutation rate to be 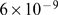, then this is equivalent to 55,784.5 generations. If the actual mutation rate in *Aquilegia* is higher than we assumed, then the estimated number of generations since the split will be lower, and if the actual mutation rate is lower, than the number of generations will be higher. The generation time in *Aquilegia* is not known, but a very rough estimate can be calculated as 10 years, based on the observation that the plants seem to produce seeds in the wild for about 20 years. If we assume the generation time is around 10 years, then the MIMAR results suggest that *A. formosa* and *A. pubescens* diverged 557,845 years ago. When migration was incorporated into the model, the estimate for the time since the split rose slightly (to 660,860 years). Both of these estimates seem reasonable, given that the diversification of the North American *Aquilegia* clade is believed to have occurred less than 2 million years ago [31].

Although we obtained believable estimates of the divergence time, MIMAR was not able to converge on an estimate for the migration rate, despite the fact that the model seemed to be mixing well and the estimates of 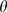 corresponded to our earlier calculations (data not shown). Using Wright's 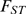
–based estimator of migration rate, we calculated that the average number of migrants between populations per generation (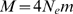) was 6. Because the MIMAR analysis suggests that *A. formosa* and *A. pubescens* have diverged recently, it is reasonable to assume that at least some of the shared variation is due to ancestral polymorphism, and is not solely the result of gene flow between the two species. Therefore, this estimate of 6 migrants per generation should be considered as a maximum possible value for 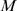.

## Discussion

We used direct sequencing to compare levels of intra- and interspecific variation in *Aquilegia*, and found that our genetic data could not distinguish *A. formosa* and *A. pubescens*. Not only were values of 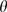 strikingly similar across species for every fragment, but estimates of 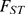 were also extremely low, indicating that almost all polymorphism is shared between species. This is a remarkable finding given that these two species are strongly differentiated both ecologically and morphologically.

Several studies of other species have uncovered the same phenomenon. Different species of wild sunflowers exhibit strong ecological differentiation, but it has been found that there are few fixed differences between the species, despite very high levels of intraspecific variation (higher than what we observed in *Aquilegia*) [4]. Hybridization also occurs between these species, and there is evidence for long-term introgression since their divergence one million years ago [4]. Gene flow has also played a role in shaping the patterns of genetic divergence among species in the Hawaiian silversword alliance, which (like *Aquilegia*) is another example of an adaptive radiation in plants [32]. Finally, African cichlid fishes represent one of the most dramatic examples of an adaptive radiation, and many of the more than 2,000 unique species in this group have arisen via sympatric speciation and are still capable of forming viable hybrid offspring, despite many ecological, morphological, and behavioral differences [33].

As in the above examples, it is known that hybrid zones form between *A. formosa* and *A. pubescens* [20]–[22]. There are also some genetic markers which suggest introgression beyond the hybrid zones [22], which makes it tempting to speculate that gene flow between the species has been occurring since their divergence. Our implementation of the isolation-migration model [34], [35] produced an estimate of the divergence time that fit well with the model of recent speciation, but since it could not simultaneously converge on an estimate for the migration rate, we cant be sure that gene flow is still occurring. This may be the result of too little data in general, or it may also be the result of having zero fixed differences in the sample.

The patterns of population structure were also unclear in our sample; geographic distance between populations has a clear correlation with genetic differentiation in the broad sample of North American *Aquilegia* taxa, but there is not a clear relationship when only *A. formosa* and *A. pubescens* are examined on a more local scale. At the same time, the clustering of individuals in STRUCTURE does not seem entirely random, with two individuals being more likely to cluster together if they are from the same species and the same population than if they are not. It is possible that these results are a reflection of a pre-exisiting population structure in the common ancestor, or that migration between populations has made the structure harder to discern.

We believe that finding the loci responsible for reproductive isolation will help us to gain a clearer understanding of how speciation has occurred in *Aquilegia*. A relatively recent scan of genome-wide patterns of interspecific differentiation in two species of European oaks led to the identification of a few genomic regions which seem to underlie species divergence [36]. Like *Aquilegia*, these oak species were closely related and highly interfertile, despite exhibiting significant differences in ecology and morphology. The overall low levels of interspecific variation in these species facilitated the identification of highly differentiated regions. The primary goal of this study was to assess the feasibility of a similar type of genome-wide scan for highly differentiated loci in *Aquilegia*. Our results have shown that despite reasonable levels of intraspecific polymorphism, genetic differentiation is incredibly low at neutral loci, which should make it easier to distinguish putative speciation genes.

## Materials and Methods

### Sample Collection and Preparation

Leaf tissue was collected from individual plants found in different locations along the west coast of North America. Samples were taken from 40 individuals of each species, for a total sample size of 80 individuals. *A. formosa* samples were taken from 9 different populations, ranging from California, Nevada, Washington state, British Columbia, and Alaska. The number of individuals in each of these populations varied between 1 and 10, but most populations had 5 individuals. There were only 3 populations of *A. pubescens*, and all of them were from California. There were between 4 and 16 individuals in each of these populations (see also Table S1 for a description of the sampling). Because the *A. pubescens* populations were less geographically dispersed than the *A. formosa* samples, there was some concern that *A. pubescens* might falsely appear to be less polymorphic than *A. formosa*. However, as was discussed in the Results section, the same level of polymorphism was found in both species, so sampling bias was not an issue.

DNA extractions were performed using Qiagen's DNeasy Plant Mini Extraction Kits. Due to limited sample amounts, extracted DNA was used directly in only 5 out of the 9 amplifications (*Acetyl*, *Defen*, *H3*, *LFY*, and *UF3GT*). For the remaining 4 amplifications, the extracted DNA was first amplified using Qiagen's REPLI-g Mini Kit and corresponding whole genome amplification protocol.

Additional leaves were collected from thirty-two *Aquilegia* taxa (including *A. formosa* and *A. pubescens*) [17]. Twenty-five of these are also native to North America, while the remaining 7 are found in Europe and Asia. For each species, between 1 and 3 populations were sampled, with an average of 5 individuals per population (Table S1). The majority of individuals came from western North America. DNA extractions were performed as described above.

### Fragment Amplification and Sequencing

Nine short regions of the *Aquilegia* genome were amplified in the original sample via PCR using 3′-UTR anchored primers (Table S2). These primers were originally designed by Whittall et al. [2] to reconstruct a species-level phylogeny for several members of the *Aquilegia* genus (including *A. formosa*, but excluding *A. pubescens*). None of these regions are expected to be involved in the evolution of reproductive barriers. Two of the 9 regions were also amplified in the broader sample of 32 species (*Gapc* and *UF3GT*). All of the sequences contained some non-exonic DNA (Table S3).

All PCR amplifications were done in a total volume of 25
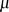
L, with 20
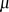
L Promega PCR Master Mix (2×: 50 units per mL of *Taq* polymerase, 400
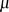
M dATP, 400
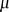
M dGTP, 400
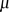
M dCTP, 400
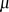
M dTTP, 3mM Mg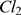), 3
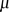
L of forward and reverse primers (10
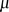
M each), and approximately 20 ng of DNA template. Although the annealing temperature varied slightly among primer pairs, the cycling conditions were generally as follows: 92

C for 2 minutes, followed by 35 cycles of: 92

C for 45 seconds, 61

C for 30 seconds, 72

C for 1.5 minutes, and a final extension step at 72

C for 10 minutes.

Sequencing for the original sample of 80 individuals was performed in both directions using the Beckman-Coulter CEQ 2000 platform. Purifications and sequencing reactions were all done as recommended by the Beckman-Coulter protocols. PCR products were purified using Promega's Wizard MagneSil PCR Clean-Up System. Eight microliters of purified template were mixed with 1
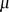
L CEQ 10× Buffer, 1
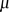
L CEQ QuickStart Mix, 2.8
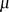
L water, and 0.25
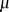
L of either forward or reverse primer (for a total reaction volume of 13
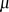
L). The sequencing reaction mixtures were then subjected to the following cycling conditions: 96

C for 20 seconds, 50

C for 20 seconds, and 60

C for 4 minutes for a total of 40 cycles, followed by holding at 4

C. The reaction products were cleaned up using the Beckman-Coulter protocol for “Ethanol Plate Precipitation in a CEQ sample plate,” and then finally loaded into the CEQ 2000 for sequencing. Sequencing for the broader sample was performed on the Li-Cor System.

### Sequence Alignment and Editing

Sequences obtained from the CEQ 2000 were aligned using phredPhrap [37], [38], and visualized in Consed [39]. All alignments were edited manually with the aid of MABCW (program written by T. Hu; scripts and more information available upon request). The indel polymorphisms that we were able to identify were all relatively short, and we only observed two alleles at each of these sites. We were not able to characterize individuals that were heterozygous at these sites, and treated these sequences as missing data during our analyses. For homozygous individuals, indels were analyzed as biallelic SNPs.

For each fragment, the set of segregating sites was identified using alignments of all sequences from both species. The sites in this set were then subsequently characterized as either exclusive to one species or shared based on whether or not they were still segregating in an alignment of sequences from only one species. At each SNP position, the derived allele was determined by using a draft assembly of the *Aquilegia coerulea* (Goldsmith) genome as an outgroup (Joint Genome Institute (JGI) *Aquilegia* Sequencing Project, unpublished data).

For the purpose of linkage disequilibrium analyses, haplotypes were reconstructed using PHASE 2.0.2 [40], [41]. For all other analyses, (estimation of 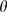, 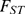, MIMAR, and population structure), we used the un-phased genotype data directly.

### Analysis

The population mutation parameter (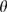) was estimated using Watterson's estimator (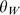) [42] and the average number of pairwise differences (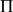) [43]. Using in-house scripts (available upon request), both of these statistics were determined for each of the 9 sets of sequences and then scaled by the length of the sequence in order to get a per base pair value. The reading frame for each fragment was assumed based on alignment with cDNA sequences available in Genbank (accession numbers: DQ286961, DQ224264, DQ224271, DQ217409, DQ286960, DQ224258, AY162852, and DQ286959). Estimates of 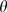 for different classes of sites were scaled by the total number of silent sites or nonsynonymous sites in each sequence (see Table S4 for results). The number of silent sites (S) and the number of nonsynonmous sites (N) were calculated based on a simple Jukes-Cantor model of substitution [44], with the following equations: 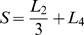, 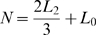, where 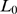 is the number of non-degenerate sites, 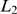 is the number of twofold degenerate sites, and 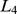 is the number of fourfold degenerate sites.

Linkage disequilibrium (LD) between SNPs was quantified using 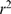, the squared correlation coefficient. For each fragment, 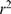 was plotted as a function of the distance between SNPs (measured in base pairs). The population recombination parameter (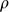) was estimated by fitting the equation given in [45], [46]:
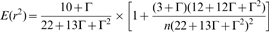
where 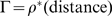. For all analyses of recombination, low frequency (MAF 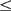
10%) polymorphisms were removed, since they provide little information about the overall pattern of LD.

Estimates of Wright's 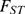 were calculated based on estimates of 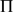 [47] using the following equation:
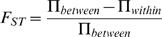
where 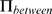 refers to the average pairwise difference between individuals from different species, and 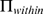 is the average pairwise difference within species. Confidence intervals were obtained by using 10,000 bootstrap replicates.

Analysis of Isolation By Distance (IBD) was performed using a Mantel test [48] with 10,000 replications as implemented by the R package “ade4” [49], [50]. The genetic distance matrix was composed of estimates for 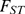 while the geographic distance matrix was measured in kilometers between populations.

Population structure was inferred directly from the sequence data using the program STRUCTURE 2.0, which implements a model-based clustering approach [51]. STRUCTURE was run under the “linkage model” with “correlated allele frequencies.” Specifying correlated allele frequencies enhances the ability of the algorithm to detect distinct clusters even among a sample of very closely related populations [52], which is well suited to the *Aquilegia* data set. Although geographic sampling information was available, initial STRUCTURE runs suggested that geographic location did not correspond well with the genetic data, so we did not use the “prior population information” model to assist in clustering. The program was run with a burn-in length of 50,000 and a run length of 20,000. This was done several times for each K value (ranging from 2 to 15) in order to ensure that results were consistent. Plots of the STRUCTURE output were generated using *distruct* [53]. The average “clusteredness” of individuals was calculated for each STRUCTURE run according to the equation presented by Rosenberg et al. [52].

In order to estimate divergence time and migration rate, the data were analyzed using the program MIMAR [35], which can incorporate recombination into an “isolation–migration” model. The mutation rate, 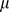, was assumed to be 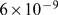, based on an estimation of the average substitution rate in nuclear DNA in plants [54]. The intralocus recombination rate was set at 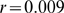, based on the estimation of the population recombination rate from linkage disequilibrium data. 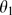, 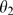, and 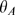 were all sampled from a uniform prior distribution 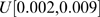. The time since split, 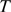, measured in generations, was sampled from the prior distribution 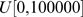. Migration was either fixed at 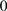, or drawn from a prior range between 0.135 and 7.39. The program was run for 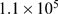 recorded steps, and 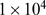 burnin steps.

We also estimated the migration rate using Wright's equation [55] for an n-island population model, which is based on 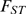:
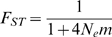



## Supporting Information

Figure S1 STRUCTURE cannot cluster individuals according to species. Each individual is indicated by a thin line, where the two colors represent the estimated membership coefficients for the 2 clusters. The clusteredness score for this plot was estimated as 0.26.
(0.04 MB PDF)

Figure S2 Inferred population structure for 80 *Aquilegia* individuals. The results from STRUCTURE are plotted for K = 11, which had an average clusteredness score of ≈0.52. Each individual is represented by a thin horizontal line, with corresponding population and species information given on either side.
(0.06 MB PDF)

Figure S3 Probability of different K estimates. The estimated log probability of the data (as calculated by STRUCTURE) is plotted against different K values. For each K value, STRUCTURE was run 3 times, and the plotted value is the average of those 3 runs.
(0.03 MB PDF)

Figure S4 Average clusteredness for different K values. For each K value, the average clusteredness measures the extent to which each individual belongs to a single cluster rather than to multiple clusters, so the higher the clusteredness the “better” the clusters.
(0.03 MB PDF)

Figure S5 Other factors influencing F_ST_ in *Aquilegia*. In all panels, red dots indicate comparisons where both populations were the same for the factor being considered, while gray dots indicate comparisons where the two populations were different. Panel (A) shows F_ST_ vs distance both within and between species, with the green diamonds indicating comparisons between either *A. formosa* or *A. pubescens* and one of the natural hybrid populations. Panel (B) shows F_ST_ vs distance with the same and different pollinator syndrome, while Panel (C) shows the same comparisons for habitat type.
(0.09 MB PDF)

Figure S6 MIMAR estimates of time since divergence
(0.41 MB PDF)

Figure S7 R^2^ versus distance for the combined data.
(0.07 MB PDF)

Table S1 Summary of *Aquilegia* samples used in this study.
(0.04 MB PDF)

Table S2 Primer pairs used to amplify the 9 nuclear loci.
(0.03 MB PDF)

Table S3 Positions of introns, exons, and UTRs in each locus.
(0.02 MB PDF)

Table S4 Levels of polymorphism for synonymous and nonsynonymous sites.
(0.04 MB PDF)
