# Supplementary material for: Genetic Variation at Nuclear Loci Fails to Distinguish Two Morphologically Distinct Species of Aquilegia
Source: PLoS One. 2010 Jan 19;5(1):e8655. doi: 10.1371/journal.pone.0008655 (PMC2808223; doi:10.1371/journal.pone.0008655)
Supplement: Figure S1 — STRUCTURE cannot cluster individuals according to species. Each individual is indicated by a thin line, where the two colors represent the estimated membership coefficients for the 2 clusters. The clusteredness score for this plot was estimated as 0.26. (0.04 MB PDF) [file pone.0008655.s001.pdf]

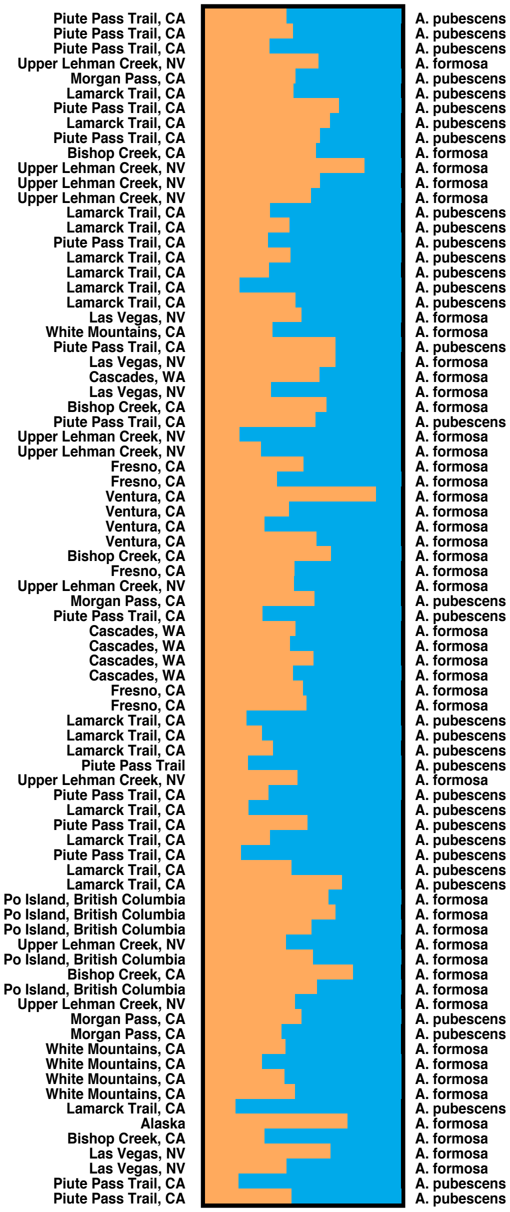

Figure S1: **STRUCTURE cannot cluster individuals according to species.** Each individual is indicated by a thin line, where the two colors represent the estimated membership coefficients for the 2 clusters. The clusteredness score for this plot was estimated as 0.26.
