# Supplementary material for: Genetic Variation at Nuclear Loci Fails to Distinguish Two Morphologically Distinct Species of Aquilegia
Source: PLoS One. 2010 Jan 19;5(1):e8655. doi: 10.1371/journal.pone.0008655 (PMC2808223; doi:10.1371/journal.pone.0008655)
Supplement: Figure S2 — Inferred population structure for 80 Aquilegia individuals. The results from STRUCTURE are plotted for K = 11, which had an average clusteredness score of ≈0.52. Each individual is represented by a thin horizontal line, with corresponding population and species information given on either side. (0.06 MB PDF) [file pone.0008655.s002.pdf]

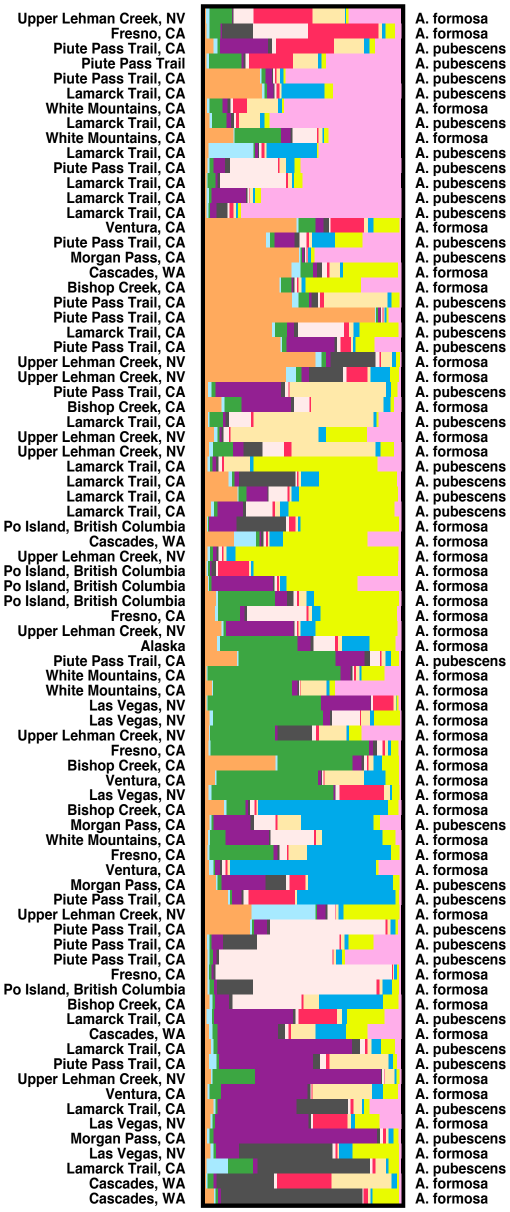

Figure S2: **Inferred population structure for 80 *Aquilegia* individuals.** The results from STRUCTURE are plotted for K=11, which had an average clusteredness score of ≈ 0.52. Each individual is represented by a thin horizontal line, with corresponding population and species information given on either side.
