# Supplementary material for: Genetic Variation at Nuclear Loci Fails to Distinguish Two Morphologically Distinct Species of Aquilegia
Source: PLoS One. 2010 Jan 19;5(1):e8655. doi: 10.1371/journal.pone.0008655 (PMC2808223; doi:10.1371/journal.pone.0008655)
Supplement: Figure S3 — Probability of different K estimates. The estimated log probability of the data (as calculated by STRUCTURE) is plotted against different K values. For each K value, STRUCTURE was run 3 times, and the plotted value is the average of those 3 runs. (0.03 MB PDF) [file pone.0008655.s003.pdf]

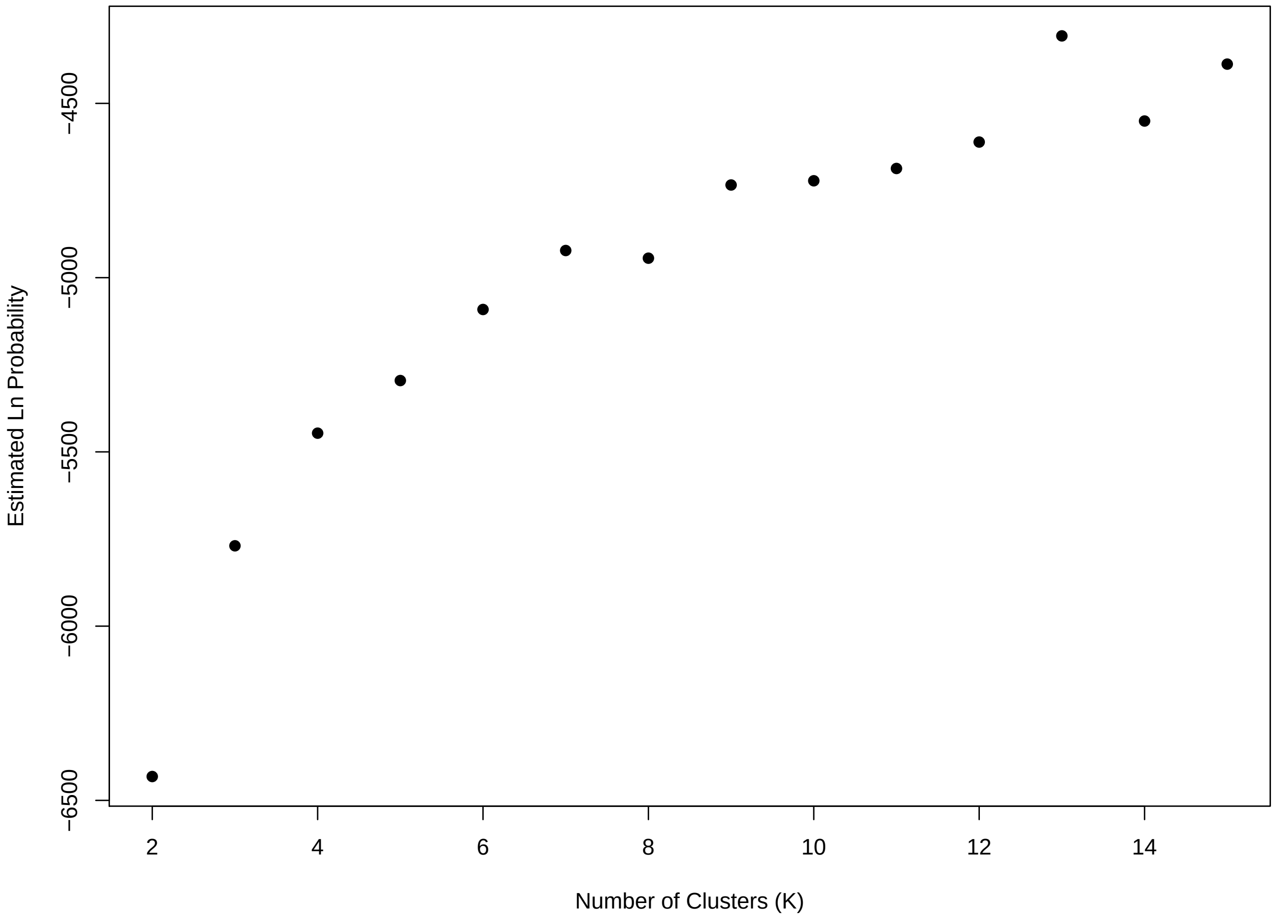

Figure S3: **Probability of Different K Estimates:** The estimated log probability of the data (as calculated by STRUCTURE) is plotted against different K values. For each K value, STRUCTURE was run 3 times, and the plotted value is the average of those 3 runs.
