# Supplementary material for: Genetic Variation at Nuclear Loci Fails to Distinguish Two Morphologically Distinct Species of Aquilegia
Source: PLoS One. 2010 Jan 19;5(1):e8655. doi: 10.1371/journal.pone.0008655 (PMC2808223; doi:10.1371/journal.pone.0008655)
Supplement: Figure S4 — Average clusteredness for different K values. For each K value, the average clusteredness measures the extent to which each individual belongs to a single cluster rather than to multiple clusters, so the higher the clusteredness the “better” the clusters. (0.03 MB PDF) [file pone.0008655.s004.pdf]

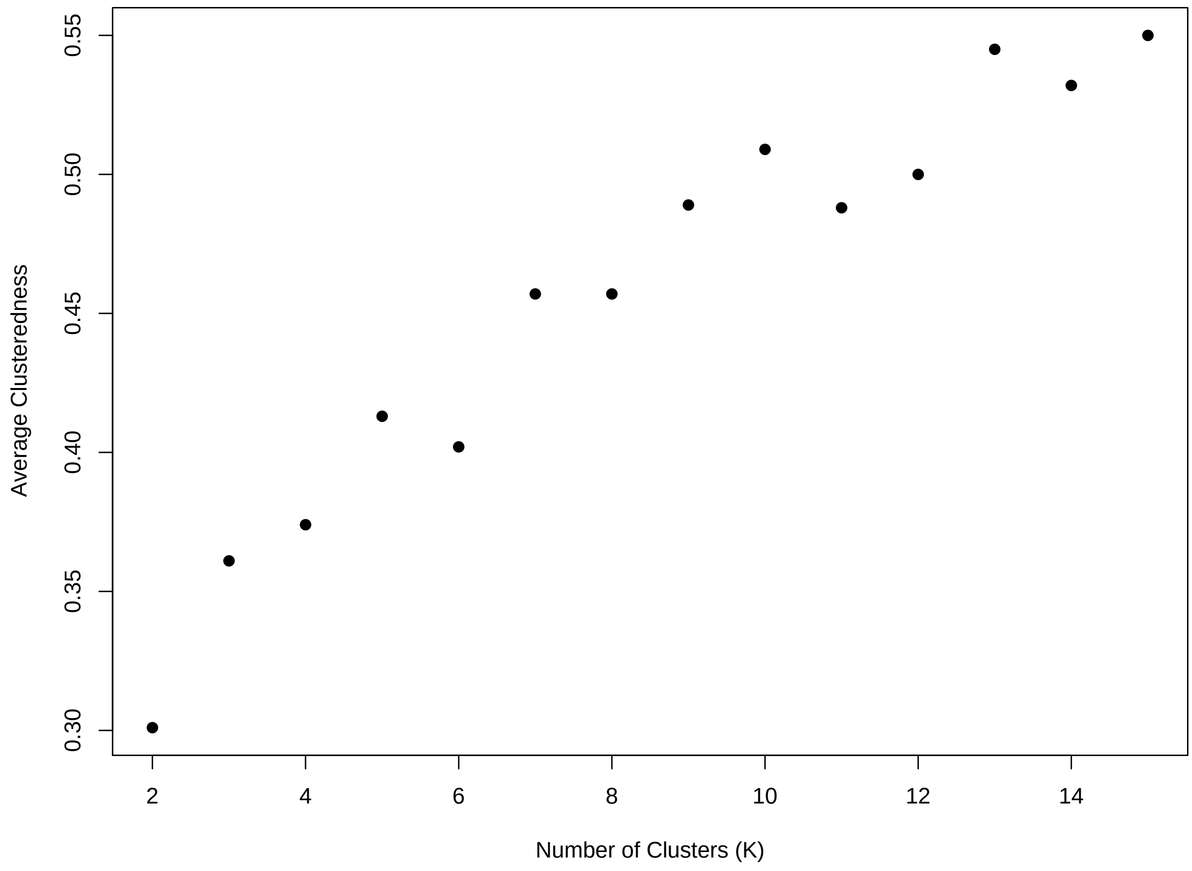

Figure S4: **Average Clusteredness for Different K Values.** For each K value, the average clusteredness measures the extent to which each individual belongs to a single cluster rather than to multiple clusters, so the higher the clusteredness the “better” the clusters.
