# Supplementary material for: Genetic Variation at Nuclear Loci Fails to Distinguish Two Morphologically Distinct Species of Aquilegia
Source: PLoS One. 2010 Jan 19;5(1):e8655. doi: 10.1371/journal.pone.0008655 (PMC2808223; doi:10.1371/journal.pone.0008655)
Supplement: Figure S5 — Other factors influencing FST in Aquilegia. In all panels, red dots indicate comparisons where both populations were the same for the factor being considered, while gray dots indicate comparisons where the two populations were different. Panel (A) shows FST vs distance both within and between species, with the green diamonds indicating comparisons between either A. formosa or A. pubescens and one of the natural hybrid populations. Panel (B) shows FST vs distance with the same and different pollinator syndrome, while Panel (C) shows the same comparisons for habitat type. (0.09 MB PDF) [file pone.0008655.s005.pdf]

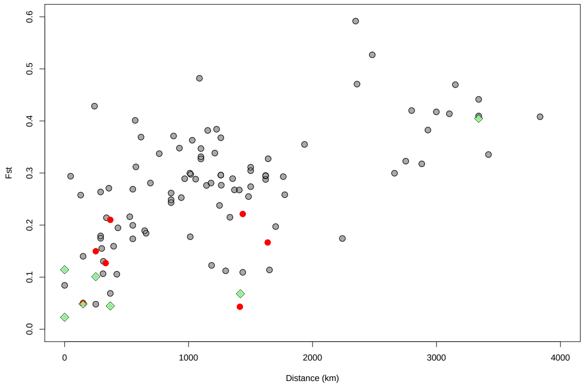

(a) Within vs. Between Species

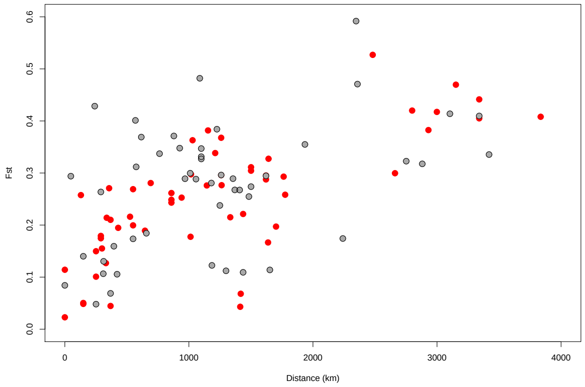

(b) Same vs. Different Pollinator Syndrome

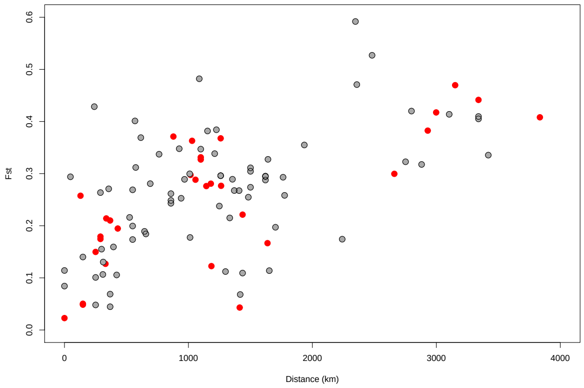

(c) Same vs. Different Habitat Type

Figure S5: **Other factors influencing F_{ST} in *Aquilegia*.** In all panels, red dots indicate comparisons where both populations were the same for the factor being considered, while gray dots indicate comparisons where the two populations were different. Panel (A) shows F_{ST} vs distance both within and between species, with the green diamonds indicating comparisons between either *A. formosa* or *A. pubescens* and one of the natural hybrid populations. Panel (B) shows F_{ST} vs distance with the same and different pollinator syndrome, while Panel (C) shows the same comparisons for habitat type.
