# Supplementary material for: Genetic Variation at Nuclear Loci Fails to Distinguish Two Morphologically Distinct Species of Aquilegia
Source: PLoS One. 2010 Jan 19;5(1):e8655. doi: 10.1371/journal.pone.0008655 (PMC2808223; doi:10.1371/journal.pone.0008655)
Supplement: Figure S6 — MIMAR estimates of time since divergence (0.41 MB PDF) [file pone.0008655.s006.pdf]

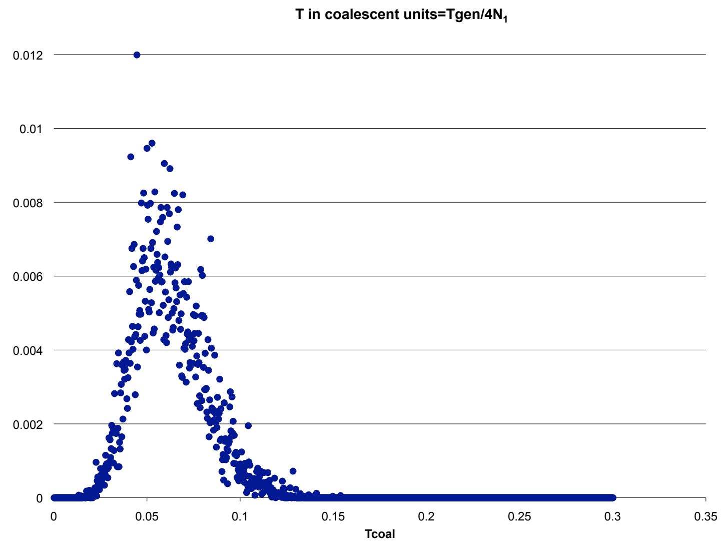

(a) No Migration

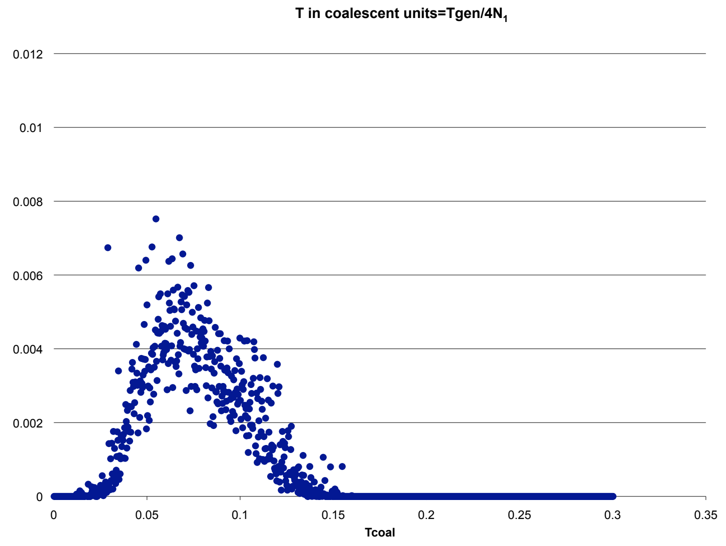

(b) With Migration

Figure S6: **MIMAR estimates of the time since divergence between *A. formosa* and *A. pubescens*.** Both panels show the posterior distributions of the time since divergence in coalescent time units. In the first panel, the distribution was generated by running MIMAR with the migration rate fixed at 0; in the second panel, the prior distribution for the migration rate was $U[0.135, 7.39]$.
