# Supplementary material for: Genetic Variation at Nuclear Loci Fails to Distinguish Two Morphologically Distinct Species of Aquilegia
Source: PLoS One. 2010 Jan 19;5(1):e8655. doi: 10.1371/journal.pone.0008655 (PMC2808223; doi:10.1371/journal.pone.0008655)
Supplement: Figure S7 — R2 versus distance for the combined data. (0.07 MB PDF) [file pone.0008655.s007.pdf]

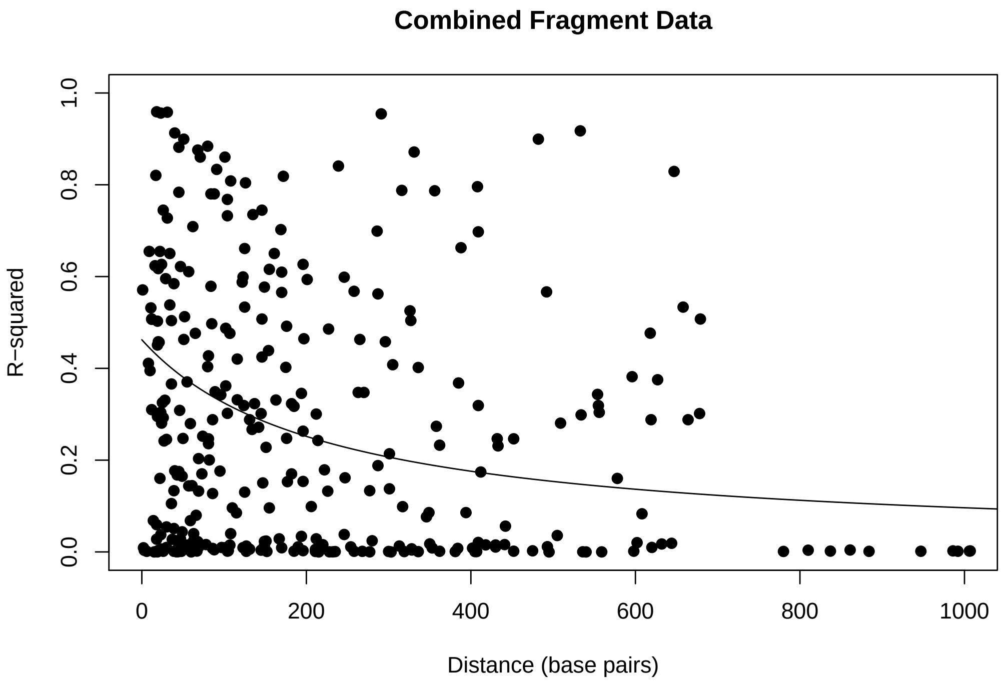

Figure S7: R^2 **versus distance for the combined data.** Only SNPs with a minor allele frequency $\geq 10\%$ were used in pairwise comparisons. Trend line was fitted as described in the Methods section.
