# Supplementary material for: Genetic Variation at Nuclear Loci Fails to Distinguish Two Morphologically Distinct Species of Aquilegia
Source: PLoS One. 2010 Jan 19;5(1):e8655. doi: 10.1371/journal.pone.0008655 (PMC2808223; doi:10.1371/journal.pone.0008655)
Supplement: Table S1 — Summary of Aquilegia samples used in this study. (0.04 MB PDF) [file pone.0008655.s008.pdf]

Table S1: Summary of *Aquilegia* samples used in this study.

Species	Population	Num. Individuals	Sample
A. barnebyi	Rifle Falls, CO	3	Second
A. brevistyla	Alberta, Canada	5	Second
A. canadensis	Kimbalton, Virginia	5	Second
A. canadensis	Ontario, Canada	5	Second
A. chaplenei	Sitting Bull Falls, NM	5	Second
A. chrysantha	Ash Canyon, NM	5	Second
A. chrysantha	El Salto, NM	4	Second
A. coerulea var. *alpina*	Jackson Hole, WY	5	Second
A. coerulea var. *coerulea*	Loveland Pass, CO	5	Second
A. coerulea var. *coerulea*	Sugarloaf, WY	5	Second
A. coerulea var. *coerulea*	Tony Grove, UT	5	Second
A. pinetorum	Sawmill, AZ	5	Second
A. desertorum	Horse Spring, AZ	1	Second
A. elegantula	Hyde Memorial, NM	5	Second
A. eximia	Porter Camp, CA	2	Second
A. flavescens	High Creek, UT	5	Second
A. formosa	Alaska	1	First
A. formosa	Bass Lake, CA	5	Second
A. formosa	Bishop Creek, CA	5	First
A. formosa	Cascades, WA	5	First
A. formosa	Fresno, CA	5	First
A. formosa	Las Vegas, NV	5	First
A. formosa	Po Island, British Columbia	5	Second
A. formosa	Po Island, British Columbia	5	First
A. formosa	Upper Lehman Creek, NV	5	Second
A. formosa	Upper Lehman Creek, NV	10	First
A. formosa	Ventura, CA	4	First
A. formosa	White Mountains, CA	5	First
A. hinckleyana	Capote Falls, TX	5	Second
A. jonesii	Medicine Wheel, WY	2	Second
A. laramiensis	Friend Camp, WY	2	Second
A. longissima	Baboquivari, AZ	4	Second
A. longissima	Maple. TX	5	Second
A. micrantha	Escalante, CO	5	Second
A. pubescens	Lamarck Trail, CA	16	First
A. pubescens	Morgan Pass, CA	4	First
A. pubescens	Piute Pass Trail, CA	5	Second
A. pubescens	Piute Pass Trail, CA	15	First

Supp. Table 1: continued

Species	Population	Num. Individuals	Sample
A. saximontana	Pikes Peak, CO	5	Second
A. scopulorum	Charleston Peak, NV	5	Second
A. shockleyi	Charleston Library, NV	5	Second
A. skinneri	Mesa del Campanero, Sonora, Mexico	5	Second
A. triternata	Zion, UT	5	Second
A. caucausica	Europe	1	Second
A. flabellata	Japan	3	Second
A. fragrans	Europe	1	Second
A. glandulosa	Asia	1	Second
A. olympica	Europe	2	Second
A. pyrenaica	Europe	2	Second
A. viridiflora	Siberia	3	Second
Hybrids (*formosa* and *pubescens*)	Lamarck Lakes, CA	11	First
Hybrids (*formosa* and *pubescens*)	Piute, CA	5	First
Sp. nov.	Stella Lakes, NV	5	Second
