# Supplementary material for: Genetic Variation at Nuclear Loci Fails to Distinguish Two Morphologically Distinct Species of Aquilegia
Source: PLoS One. 2010 Jan 19;5(1):e8655. doi: 10.1371/journal.pone.0008655 (PMC2808223; doi:10.1371/journal.pone.0008655)
Supplement: Table S2 — Primer pairs used to amplify the 9 nuclear loci. (0.03 MB PDF) [file pone.0008655.s009.pdf]

Table S2: Primer pairs used to amplify the 9 nuclear loci

Locus	Fragment Size (bp)	Primers (Forward=top; Reverse=bottom)
Acetyl-CoA carboxylase (Acetyl)	577	ATTCGCGGAGCTACATGATA<br>CCTACTGCTACTTTCAACAATCAAC
Defensin protein (DEFEN)	683	GCAACATGCGTCTAGTTTCAG<br>GAACCACGAAGGTGACCCT
Glyceraldehyde-3-phosphate dehydrogenase (GAPC)	1097	GTCTGAGGGCAAACTGAAGG<br>AAACCTGAAGCAGCAATAGGA
Histone H3	358	CAAACTTCCCTTCCAACGTC<br>AACTTCCGATATATTTCATTCATTG
HSP70-1 (HEAT)	1281	TCTTCAGGGAGAGAGAGAGTTTG<br>ATTACTTCCCCACCATCAGG
Apetala-III (AP3)	1093	TGAGTCTGTGAAACTTGTTCGGG<br>GCAATGCGAATAGCAATGCC
LFY	575	CCCAACCAAGGTATGAATA<br>CCTGAATTGCATGTCGATACAC
Pistalla-AqaPI-1 (Pist)	1200	CGAACTCAGGCACTTGAAGG<br>GCATTGTTGAATGTTGATACACTCT
Glycosyl transferase (UF3GT)	473	GAGGAAGCTTTGCCAGAGG<br>AAATGCGACACTGCGACATA
