# Supplementary material for: Genetic Variation at Nuclear Loci Fails to Distinguish Two Morphologically Distinct Species of Aquilegia
Source: PLoS One. 2010 Jan 19;5(1):e8655. doi: 10.1371/journal.pone.0008655 (PMC2808223; doi:10.1371/journal.pone.0008655)
Supplement: Table S3 — Positions of introns, exons, and UTRs in each locus. (0.02 MB PDF) [file pone.0008655.s010.pdf]

Table S3: Positions of introns, exons, and UTRs in Each Locus

Locus	Exon Positions	Intron Positions	UTR Positions
Acetyl	1...389, 485...542	390...484	543...577
DEFEN	1...38, 389...560	39...388	561...683
GAPC	1...59, 231...318, 844...888	60...230, 319...843	889...1097
H3	1...200	...	201...358
HEAT	1...192, 477...482, 930...773	193...476, 483...929	774...1281
AP3	1...6, 120...163, 542...587, 678...832	7...119, 164...541, 588...677	833...1093
LFY	...	1...575	...
Pist	1...762	763...945	946...1200
UF3GT	1...424	...	425...473
