# Supplementary material for: Genetic Variation at Nuclear Loci Fails to Distinguish Two Morphologically Distinct Species of Aquilegia
Source: PLoS One. 2010 Jan 19;5(1):e8655. doi: 10.1371/journal.pone.0008655 (PMC2808223; doi:10.1371/journal.pone.0008655)
Supplement: Table S4 — Levels of polymorphism for synonymous and nonsynonymous sites. (0.04 MB PDF) [file pone.0008655.s011.pdf]

Table S4: Levels of polymorphism for synonymous and nonsynonymous sites

	Synonymous				Nonsynonymous			
	θ_W		Π		θ_W		Π	
Fragment	*A.f*	*A.p*	*A.f*	*A.p*	*A.f*	*A.p*	*A.f*	*A.p*
Acetyl	0.0010	0.0011	0.0013	0.0013	0.0013	0.0022	0.0012	0.0009
AP3	0.0037	0.0041	0.0022	0.0017	0	0	0	0
DEFEN	0.0046	0.0034	0.0026	0.0021	0	0.0079	0	0.0015
Gapc	0.0061	0.0049	0.0018	0.0014	0.0118	0.010	0.0016	0.0042
H3	0.0069	0.0087	0.0015	0.0021	0.0092	0.0081	0.0029	0.0044
Heat	0.0063	0.0073	0.0038	0.0043	0.0017	0.0035	0.0006	0.0012
Pist	0.0023	0.0009	0.0003	0.0001	0.0010	0	0.0002	0
UF3GT	0.0215	0.0211	0.0053	0.0047	0.0109	0.0101	0.0055	0.0043

Note that *LFY* is not listed in this table because it did not contain any nonsynonymous sites.
